# Deep learning-based craniosynostosis classification via suture segmentation and mask-weighted classification

**DOI:** 10.3389/fmed.2026.1871961

**Published:** 2026-08-14

**Authors:** Doheyon Park, Yong Uk Jung, Byung Jun Kim, Jisang Yoo, Jeehyeok Chung, Sungmi Jeon, Hyeyeon Kwon, Soonchul Kwon, Kang Young Choi, Jinyong Shin, Il-Hyung Yang

**Affiliations:** 1 Department of Electronic Engineering, Kwangwoon University, Seoul, Republic of Korea; 2 Department of Plastic and Reconstructive Surgery, Seoul National University College of Medicine, Seoul, Republic of Korea; 3 Graduate School of Smart Convergence, Kwangwoon University, Seoul, Republic of Korea; 4 Department of Plastic and Reconstructive Surgery, Kyungpook National University School of Medicine, Daegu, Republic of Korea; 5 Department of Plastic and Reconstructive Surgery, Jeonbuk National University Medical School, Jeonju, Republic of Korea; 6 Research Institute of Clinical Medicine, Biomedical Research Institute, Jeonbuk National University Hospital, Jeonju, Republic of Korea; 7 Department of Orthodontics, Dental Research Institute, Seoul National University School of Dentistry, Seoul, Republic of Korea

**Keywords:** craniosynostosis, deep learning, Grad-CAM, skull X-ray, suture segmentation

## Abstract

**Introduction:**

Early diagnosis of craniosynostosis (CSO) is critical to preventing neurological complications, yet skull X-ray interpretation remains subjective, and existing deep learning models often rely on secondary cranial deformations rather than the primary pathology.

**Methods:**

To address this limitation, we propose an Integrated Suture Segmentation and Classification Pipeline that explicitly learns suture information to enhance anatomical validity and diagnostic accuracy. We constructed a balanced dataset of 1,088 skull X-ray images from 368 unique patients and developed a segmentation model to identify coronal, sagittal, and lambdoid sutures. Crucially, we introduced a Mask-weighted 4-channel Input strategy, utilizing predicted suture probability maps as weights to guide the classification model's attention toward suture regions.

**Results:**

Experimental results demonstrated that the proposed method with a DenseNet-161 backbone achieved an image-level Accuracy of 0.925 and an Area Under the Receiver Operating Characteristic curve (AUROC) of 0.980. Furthermore, exam-level diagnosis via multi-view aggregation significantly improved performance, yielding an Accuracy of 0.941 and an AUROC of 0.994. Gradient-weighted Class Activation Mapping (Grad-CAM) analysis demonstrated that the model's attention is primarily directed toward specific suture lines rather than global skull shape, suggesting that the model prioritizes anatomical features over secondary deformations commonly seen in conditions like positional plagiocephaly.

**Discussion:**

This study presents a clinically interpretable and high-performance deep learning framework, highlighting its potential as a robust computer-aided referral decision support tool for primary care settings, facilitating timely specialist assessment while minimizing the need for unnecessary radiation-intensive CT scans.

## 1 Introduction

Craniosynostosis (CSO) is a congenital cranial deformity characterized by the premature fusion of one or more cranial sutures. It can lead to serious neurological and functional complications, such as increased intracranial pressure, cognitive developmental delay, and visual impairment (1). Early diagnosis within the first few months of life is critical, as timely intervention allows for highly effective and minimally invasive endoscopic surgeries (2). While Computed Tomography (CT) is the gold standard for definitive diagnosis, the associated ionizing radiation risks make skull radiography a preferred, cost-effective first-line screening tool for infants (3). However, interpreting skull X-rays is highly subjective and challenging due to the low resolution and complex superimposition of fine suture lines, which often vary significantly depending on the projection angle.

To address these diagnostic challenges, recent studies have developed automated screening models utilizing deep learning on skull X-rays (4, 5) or emerging radiation-free modalities such as 3D photogrammetry and 2D clinical photographs (6–8). While these previous studies have already achieved expert-level diagnostic performance, Gradient-weighted Class Activation Mapping (Grad-CAM) (9) analyses suggest that these models predominantly rely on secondary external morphological deformations (e.g., facial bones, nasal cavity, global skull shape). From a clinical perspective, these secondary signs require careful differentiation as they can share morphological similarities with non-pathological conditions such as positional plagiocephaly (10). According to Virchow's Law as redefined by Delashaw al., even if cranial deformation occurs, the primary pathology, suture fusion, may not necessarily be present (10, 11).

To overcome these limitations and enhance anatomical validity, we propose an integrated deep learning framework that explicitly localizes and learns the suture information directly from skull X-rays. A segmentation-based classification framework has already proven highly effective in other medical imaging applications, such as COVID-19 diagnosis using chest X-rays, by successfully guiding models to focus on essential anatomical regions (12). This study extends this concept to cranial sutures. By employing a Mask-weighted 4-channel Input strategy, our pipeline utilizes automatically segmented coronal, sagittal, and lambdoid suture probability maps to guide the classification model's attention. This approach offers an accessible, cost-effective, and interpretable computer-aided referral decision support tool for primary care settings, assisting general practitioners in identifying suspicious cases to facilitate timely referral.

In summary, previous skull X-ray deep learning studies have achieved excellent diagnostic performance; however, Grad-CAM analyses indicate that these models primarily rely on secondary cranial deformations rather than the primary pathology of suture fusion. According to Virchow's law, cranial deformation may occur without true suture fusion, making differentiation from conditions such as positional plagiocephaly clinically challenging. Therefore, explicitly localizing and learning the cranial sutures may improve the anatomical validity and clinical reliability of AI-assisted diagnosis.

## 2 Materials and methods

### 2.1 Dataset and pre-processing

This work involved human subjects and was approved by the Institutional Review Board (IRB) of Seoul National University Hospital under IRB No. 2505-035-1638. The study was conducted under the protocol titled “Development of an AI-based Program Using Skull Radiographs for Early Diagnosis of Craniosynostosis.”

A total of 1,088 skull X-ray images were collected in this study. Following the removal of duplicate radiographs acquired across multiple views and longitudinal follow-ups, the dataset comprised a total of 368 unique patients. The Normal group consisted of 232 patients (mean age: 59.8 ± 32.9 months; 69.4% male), while the Craniosynostosis group included 136 patients (mean age: 15.0 ± 18.5 months; 56.2% male). Given the retrospective, single-center nature of the data collection in the Republic of Korea, all included patients were of a single race/ethnicity (Asian/Korean). Detailed patient demographics are summarized in Table 1.

**Table 1:** Patient demographics.

Characteristic	Normal (*n* = 232)	Craniosynostosis (*n* = 136)	Total (*n* = 368)
Age (months)	59.8 ± 32.9	15.0 ± 18.5	42.5 ± 35.6
Age (years)	4.98 ± 2.74	1.25 ± 1.54	3.54 ± 2.97
Sex, *n* (%)			
Male	161 (69.4%)	82 (56.2%)	243 (64.3%)
Female	71 (30.6%)	64 (43.8%)	135 (35.7%)

Furthermore, the number of radiographic views acquired per patient varied. The average number of views per examination was 2.22 overall, with the Craniosynostosis group averaging 3.28 views and the Normal group averaging 1.61 views. This difference is not an intended bias but rather reflects the strict adherence to the ALARA (As Low As Reasonably Achievable) principle in pediatric radiography (13). Normal infants typically present for localized symptoms such as facial lacerations or head trauma, requiring only minimal projections (typically 1–2 views) to minimize radiation exposure. Conversely, patients with suspected craniosynostosis require a comprehensive evaluation of all cranial sutures, necessitating multiple projections (up to four views) in accordance with clinical guidelines.

In the collected dataset, the craniosynostosis cases can be categorized into 16 granular diagnostic subtypes based on the specific combination of fused sutures. To provide consistent criteria, we defined seven major pathological types based on the involved sutures: Coronal(Lt), Coronal(Rt), Coronal(Bi), Lambdoid(Lt), Lambdoid(Rt), Lambdoid(Bi), and Sagittal. A Normal class was also designated. Complex cases where two or more sutures are fused simultaneously are denoted by connecting the fused sutures using the ampersand (&) symbol.

The detailed composition of the dataset according to these subtypes is summarized in Table 2, which provides the number of images by diagnostic type and view. Among the views, the Lat(Rt) view accounts for the largest portion with 447 images (41.1%), followed by AP (275 images, 25.3%), Lat(Lt) (206 images, 18.9%), and Towne's (160 images, 14.7%). Overall, the lateral views (Lat(Lt) and Lat(Rt)) constitute approximately 60.0% of the dataset. In terms of clinical prevalence, cases involving coronal suture fusion accounted for the largest proportion, comprising 316 images (53.8%). Sagittal suture fusion was present in 285 images (48.6%), and lambdoid suture fusion in 125 images (21.3%).

**Table 2:** Distribution of skull X-ray images by diagnostic type and view.

Diagnostic type	AP	Lat(Lt)	Lat(Rt)	Towne's	Total
**Normal**	**106**	**70**	**288**	**37**	**501**
Craniosynostosis subtypes					
Coronal(Bi)	43	32	43	27	145
Coronal(Bi) & Lambdoid(Bi)	3	2	3	1	9
Coronal(Bi) & Sagittal	11	10	10	9	40
Coronal(Bi) & Sagittal & Lambdoid(Bi)	3	3	3	3	12
Coronal(Lt)	4	3	4	3	14
Coronal(Lt) & Lambdoid(Bi)	1	0	1	1	3
Coronal(Lt) & Sagittal	1	1	1	1	4
Coronal(Lt) & Sagittal & Lambdoid(Bi)	1	1	1	1	4
Coronal(Rt)	21	21	20	15	77
Coronal(Rt) & Lambdoid(Rt)	2	1	1	0	4
Coronal(Rt) & Sagittal	1	1	1	1	4
Lambdoid(Rt)	13	12	13	12	50
Sagittal	53	40	46	39	178
Sagittal & Lambdoid(Bi)	9	7	9	7	32
Sagittal & Lambdoid(Lt)	2	1	2	2	7
Sagittal & Lambdoid(Rt)	1	1	1	1	4
**Subtotal (craniosynostosis)**	**169**	**136**	**159**	**123**	**587**
**Grand total**	**275**	**206**	**447**	**160**	**1,088**

Bold values represent category subtotals and the grand total of the dataset.

Systematically defining and presenting this detailed 16-class sub-classification serves two critical analytical purposes. First, it demonstrates that our dataset comprehensively covers the extensive clinical diversity and anatomical complexity of real-world cases, including various complex multi-suture fusions. Second, tracking the inherent data imbalance across these granular subtypes provides a solid foundation for clinically interpreting the model's behavior; specifically, it helps explain the dataset-driven attention bias observed in the subsequent Grad-CAM analysis (Section 3.4).

**Figure 1:**
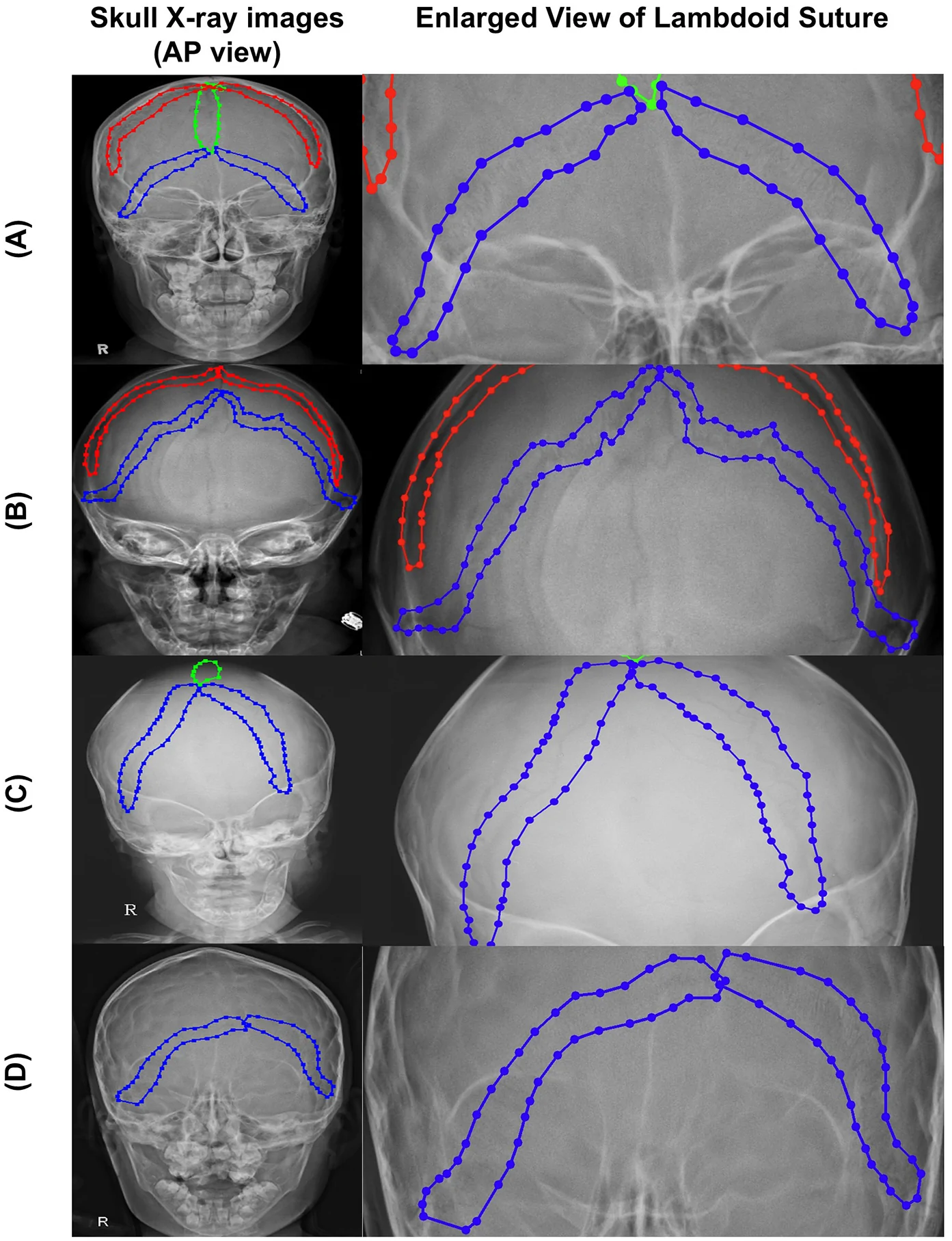
Skull X-ray images (AP view) and enlarged views of the lambdoid suture for four diagnostic types. **(A)** Normal. **(B)** Sagittal. **(C)** Coronal(Bi). **(D)** Coronal(Bi) and Sagittal.

Understanding the visual characteristics of sutures across different views is crucial for robust dataset construction and model training. Figure 1 presents skull X-ray images acquired in the Anterior-Posterior (AP) view alongside enlarged views of the lambdoid sutures. A comparison between the Normal and Sagittal types reveals that even the same suture type can appear significantly different in length and curvature depending on the child's head angle or posture at the time of imaging. In the enlarged regions, the sutures appear as fine lines with radiodensity similar to surrounding structures, making accurate annotation challenging.

**Figure 2:**
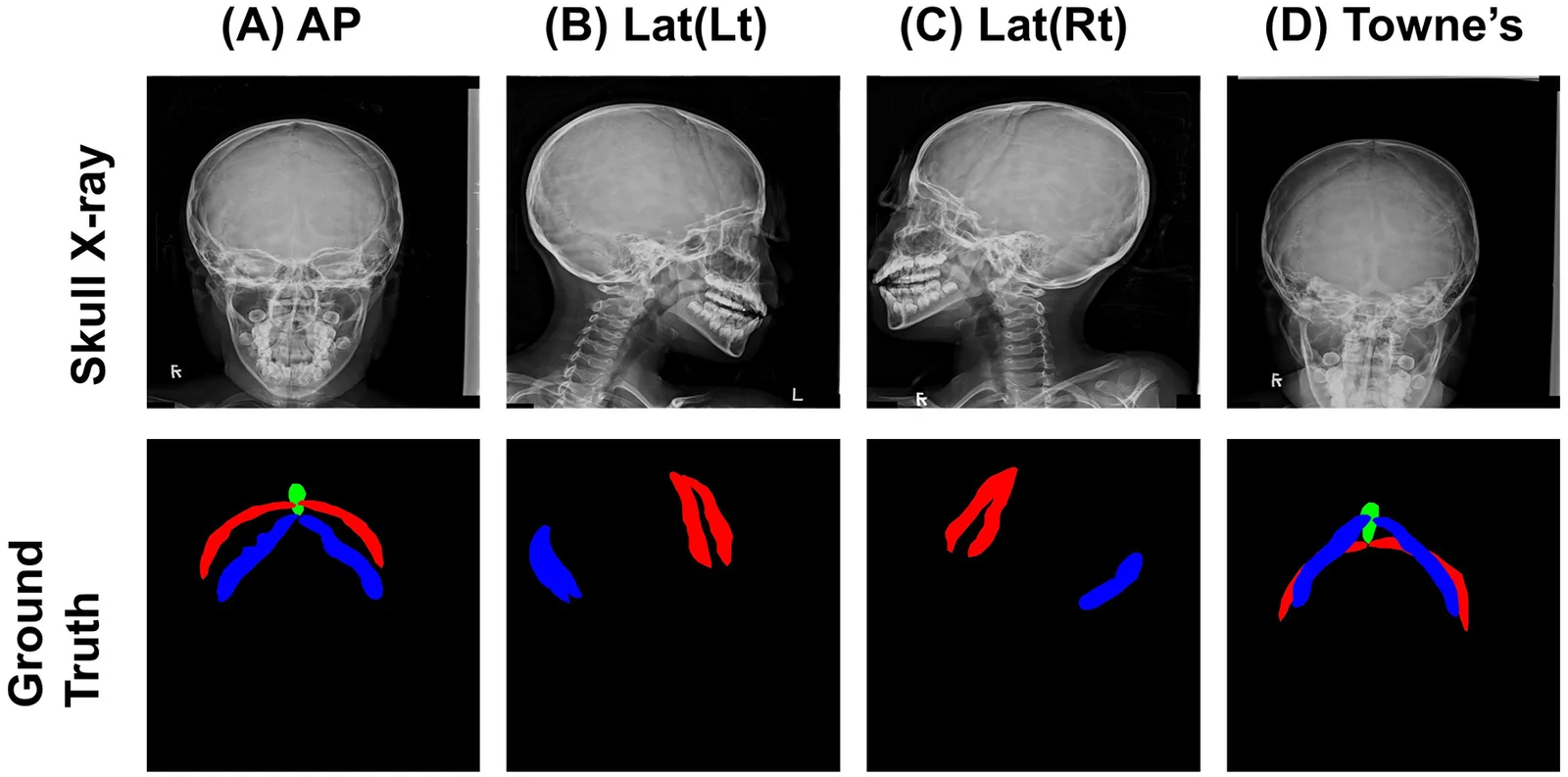
Ground-truth segmentation masks for four views of a skull X-ray from a Normal case. **(A)** AP view. **(B)** Lat(Lt) view. **(C)** Lat(Rt) view. **(D)** Towne's view.

Furthermore, a comprehensive evaluation of multiple views is essential, as the observable sutures differ by projection angle. Figure 2 displays four standard views of a Normal case along with the corresponding ground-truth segmentation masks. For instance, the AP and Towne's views capture the sagittal, coronal, and lambdoid sutures, whereas the lateral views primarily display only the coronal and lambdoid sutures. Notably, in the lateral views (Lat(Lt) and Lat(Rt)), the sutures on the left and right sides of the skull tend to visually overlap, complicating the distinction without multi-view aggregation.

The dataset was partitioned at the patient level into a training/validation set (875 images from 294 patients) and a hold-out test set (213 images from 74 patients). For model training and validation, we employed a 5-fold cross-validation scheme on the training/validation set. Crucially, to prevent data leakage, the splitting was strictly performed on a patient-level basis, ensuring that all images belonging to the same patient were assigned exclusively to a single subset. Furthermore, the split was stratified to preserve the ratio of Normal to Craniosynostosis cases across both sets.

All ground-truth annotations for the skull X-ray dataset were independently performed by two board-certified pediatric plastic surgeons with substantial diagnostic experience in craniosynostosis, and finalized through a two-step process involving mutual review. The labeling process involved manually placing multiple annotation points along the sutures in the skull X-ray images. Notably, the metopic suture often closes physiologically within a few months after birth, and isolated closure without external deformities is not considered pathological craniosynostosis (1). Accordingly, the metopic suture was explicitly excluded from the labeling and segmentation targets in this study, focusing exclusively on the coronal, sagittal, and lambdoid sutures. In particular, for patients with craniosynostosis, 3D reconstructions derived from simultaneously acquired CT scans were cross-referenced to resolve ambiguities and precisely identify the suture locations on the skull X-rays, thereby enhancing labeling accuracy.

The skull X-ray images underwent several pre-processing steps before being fed into the model to enhance image quality and improve training stability. A YOLOv8n model (14) was employed to detect both large text artifacts and the skull region, while smaller text was identified using Optical Character Recognition (OCR). All detected text regions were removed using inpainting based on surrounding pixel values. Subsequently, the images were cropped to the skull region based on the bounding boxes predicted by YOLOv8n to eliminate unnecessary background. This pre-processing procedure was adapted from a previous study (4).

The pre-processed skull X-ray images were standardized using Z-score normalization. To prevent overfitting and enhance generalizability, standard data augmentation strategies were applied, including random rotation and Gaussian noise injection.

**Figure 3:**
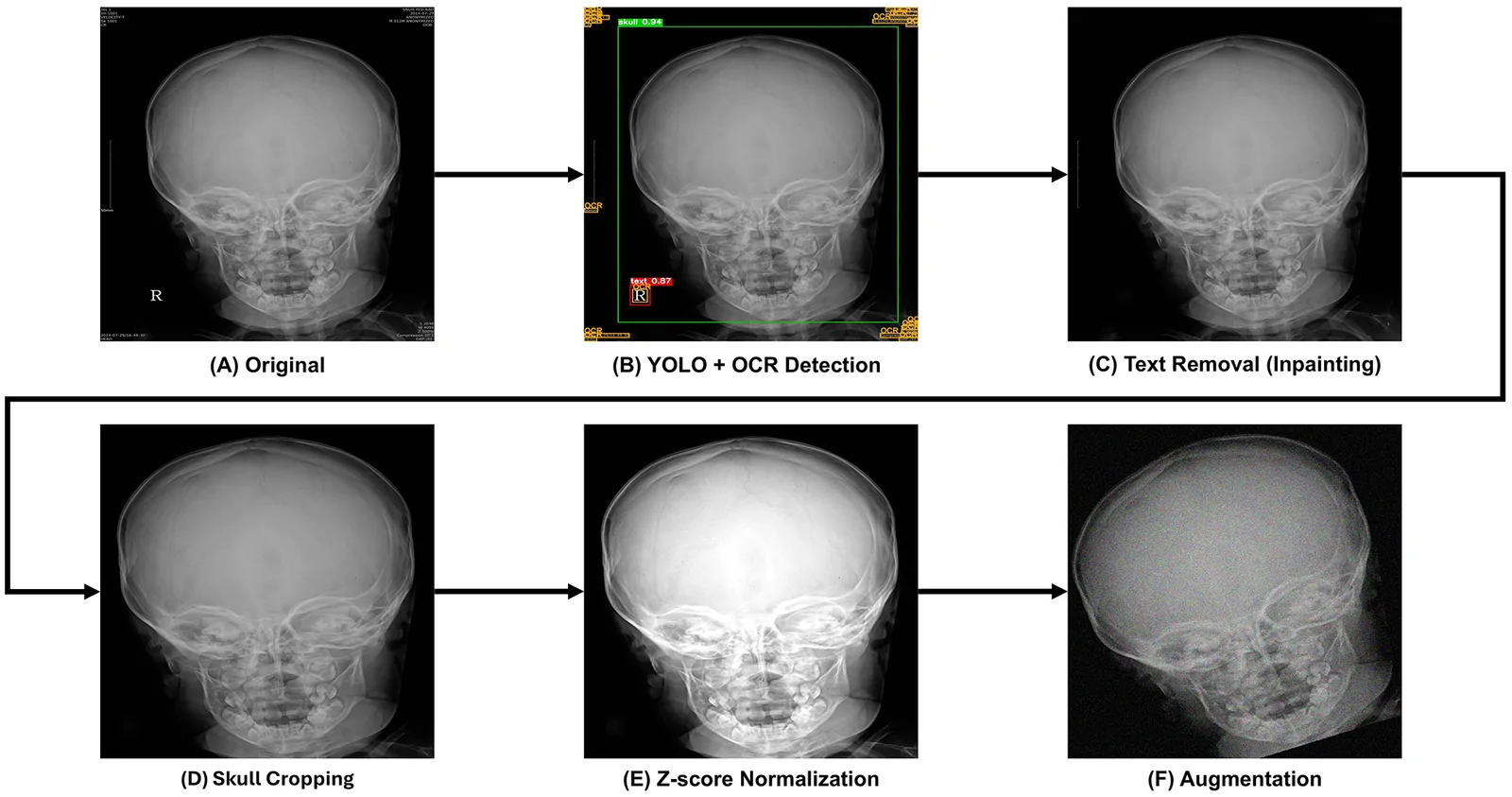
Sequential pre-processing pipeline for skull X-ray images. The process includes **(A)** Original input image, **(B)** YOLO + OCR detection for the skull and text regions, **(C)** Text removal via inpainting, **(D)** Skull cropping to eliminate the background, **(E)** Z-score normalization, and **(F)** Data augmentation. This sequence clearly visualizes the actual transformation into the final pre-processed input provided to the model.

Figure 3 sequentially illustrates the overall pre-processing pipeline applied to the skull X-ray images, visually demonstrating how the raw input is transformed into the final augmented image provided to the segmentation and classification models.

### 2.2 Overall pipeline of the proposed method

**Figure 4:**
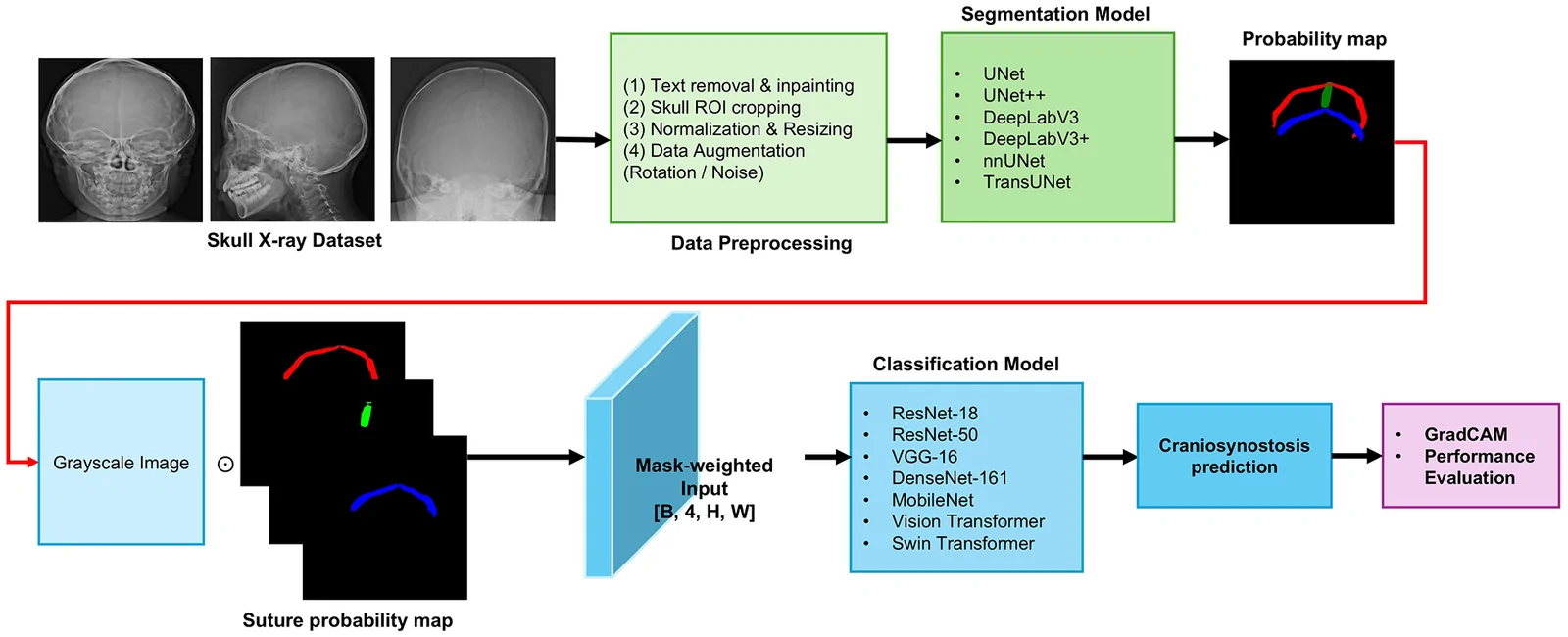
Schematic diagram of the proposed Integrated Suture Segmentation and Classification Pipeline.

Figure 4 illustrates the overall pipeline of the proposed method. Following the pre-processing phase described in the previous section, the skull X-ray images are fed into the segmentation model to generate probability maps for the coronal, sagittal, and lambdoid sutures.

Subsequently, a Mask-weighted 4-Channel Input is constructed for the classification task. The original grayscale image is replicated to form a 4-channel tensor. Three of these channels are element-wise multiplied by the predicted probability maps of the coronal, sagittal, and lambdoid sutures, respectively, while the fourth channel preserves the original image information. This composite input is then fed into the classification model to predict craniosynostosis. Finally, Grad-CAM is utilized to provide visual explainability of the model's decision, and standard metrics are employed to evaluate classification performance.

### 2.3 Segmentation model

To identify the optimal segmentation architecture for suture segmentation, we evaluated various representative models. Given the relatively small scale of the dataset and the precise nature of suture boundaries, our primary focus was on established Convolutional Neural Network (CNN)-based architectures.

We employed U-Net (15) as the standard baseline due to its symmetric encoder–decoder structure, which has proven effective for medical imaging. To compare models with enhanced feature connectivity and multi-scale contextual understanding, we included U-Net++ (16), which utilizes nested skip connections, and the DeepLab family (DeepLabV3 (17) and DeepLabV3+ (18)), which leverages atrous spatial pyramid pooling. Additionally, we utilized nnU-Net (19), a self-configuring framework that automatically adapts the network topology to dataset characteristics, serving as a robust CNN baseline.

In addition to these CNN-based models, we also adopted TransUNet (20) to evaluate the applicability of Transformer-based architectures in this specific domain. TransUNet employs a hybrid architecture that combines CNN feature maps with Vision Transformer (ViT) (21) encoders to capture long-range dependencies. This inclusion allows us to verify whether the global context modeling of Transformers offers any advantage over standard CNNs in suture segmentation tasks.

### 2.4 Mask-weighted 4-channel input

A previous study by Sharma et al. (12) successfully demonstrated a segmentation-based classification framework in COVID-19 diagnosis using chest X-ray images, achieving high accuracy by guiding the model to focus exclusively on the segmented lung regions. However, their approach relies entirely on a binary mask for a single class (the lung), which has a relatively simple anatomical structure and consistent imaging views.

In contrast, this study involves multi-class segmentation to simultaneously identify three anatomically distinct sutures across diverse X-ray views. Furthermore, due to strict patient privacy regulations and the scarcity of pediatric skull X-ray datasets, achieving perfect segmentation is extremely challenging. Under such data-constrained conditions, sole reliance on explicitly cropped masks can lead to unstable classification performance.

To overcome this limitation, this study proposes a method that first predicts probability maps for each suture and then combines them with the original image into a 4-channel input to feed into the classification model. This structure not only provides robustness against imperfect segmentation results, but also enables the model to simultaneously incorporate the probabilistic presence information of each suture.

The proposed Mask-weighted 4-channel Input uses the predicted suture probability maps from the segmentation model to weight the original grayscale image by each suture and then concatenates the resulting images together with the original grayscale image along the channel axis to form the input to the classification model.

The output of the segmentation model consists of logits for four classes: background, coronal, sagittal, and lambdoid. Using a softmax function, probability maps are obtained for each class. Among them, the probability maps for the three classes excluding the background are denoted as **S**∈ℝ^3 × *H*×*W*^ and defined as follows:


$$\begin{array}{c} \text{S}=\mathrm{Softmax}{\left(f_{\text{s}\text{e}\text{g}}\left(X\right)\right)}_{1:3}. \end{array}$$ (1)

In Equation 1, *f*_seg_(*X*) refers to the output logits of the segmentation model for the input image *X*, and the subscript 1:3 indicates the channels corresponding to the coronal, sagittal, and lambdoid classes.

Next, the original grayscale image $X_{\text{g}\text{r}\text{a}\text{y}}\in {\mathbb{R}}^{1\times H\times W}$ is multiplied element-wise with each probability map to generate the Mask-weighted images. The Mask-weighted image for class *c*, denoted as $X_{\text{m}\text{a}\text{s}\text{k}}^{\left(c\right)}\in {\mathbb{R}}^{1\times H\times W}$, is defined as:


$$\begin{array}{c} X_{\text{m}\text{a}\text{s}\text{k}}^{\left(c\right)}=X_{\text{g}\text{r}\text{a}\text{y}}⊙{\text{S}}^{\left(c\right)}. \end{array}$$ (2)

In Equation 2, *c* denotes one of the coronal, sagittal, or lambdoid classes, and ⊙ represents element-wise multiplication. In other words, each class-specific probability map is multiplied with the original grayscale image to generate a Mask-weighted image in which the corresponding suture region is emphasized.

Finally, the Mask-weighted 3-channel image *X*_mask_, formed by stacking $X_{\text{m}\text{a}\text{s}\text{k}}^{\left(c\right)}$ for all three classes, is concatenated with the original grayscale image *X*_gray_ along the channel axis to generate the final input *X*_cls_:


$$\begin{array}{c} X_{\text{c}\text{l}\text{s}}=\left[X_{\text{m}\text{a}\text{s}\text{k}};X_{\text{g}\text{r}\text{a}\text{y}}\right]\in {\mathbb{R}}^{4\times H\times W}. \end{array}$$ (3)

In Equation 3, [·;·] denotes concatenation along the channel axis. The reason for including the original grayscale image together with the segmentation maps is to preserve the original information in case the segmentation masks are incomplete. In this study, the final classification input is constructed through the procedures defined in Equations 1–3.

### 2.5 Classification model

The classification models take the proposed Mask-weighted 4-channel input to predict the presence of craniosynostosis. We evaluated both CNN-based and Transformer-based models to compare their effectiveness in feature extraction.

For CNN-based architectures, we employed ResNet-18/50 (22) and VGG-16/19 (23) as standard baselines representing residual learning and deep convolutional structures, respectively. We also included DenseNet-161 (24) to test the benefit of feature reuse through dense connectivity, and MobileNet (25) to evaluate performance under computationally efficient constraints.

For Transformer-based architectures, we utilized Vision Transformer (ViT) (21) to capture global long-range dependencies via self-attention mechanisms. Additionally, Swin Transformer (26) was adopted to leverage its hierarchical structure and shifted-window attention, which effectively balances local and global feature representation.

### 2.6 Training and implementation details

The model training consists of two stages. First, the segmentation model is trained independently, and subsequently, the classification model is trained while keeping the segmentation model's weights frozen. Both stages utilize the Adam optimizer with a learning rate of 1 × 10^−4^ and a weight decay of 1 × 10^−4^, and the learning rate is gradually decayed using a cosine annealing scheduler. The segmentation model was trained for 200 epochs with a batch size of eight, while the classification model was trained for 50 epochs with the same batch size. All input images were resized to 512 × 512 pixels and normalized using Z-score normalization computed over the entire training set (μ = 66.99, σ = 64.60). All experiments were conducted on an NVIDIA RTX 5090 (32 GB VRAM) environment using PyTorch (PyTorch Foundation, San Francisco, CA, USA) and CUDA 12.9 (NVIDIA Corporation, Santa Clara, CA, USA). To ensure reproducibility, a fixed random seed of 42 was applied to all stochastic operations, including data splitting, model initialization, and data augmentation.

Due to the significant domain discrepancy between natural images and medical X-rays, combined with the modified 4-channel input structure, transferring ImageNet-pretrained weights provides limited benefit. Thus, all classification models were randomly initialized and trained from scratch.

Model evaluation was performed using 5-fold cross-validation, where patients were assigned to folds via stratified round-robin allocation across suture types to maintain class balance. The dataset was split at the patient level to prevent data leakage, yielding 875 images (302 patients) for cross-validation and a hold-out test set of 213 images that was never used during training or fold construction. For suture fusion classification, a sigmoid threshold of 0.5 was applied, which is the standard decision boundary; this value was not tuned on the test set.

At inference, the final prediction was obtained by soft-voting across the 5 fold models, and an exam-level diagnosis was determined using an OR rule: an examination was classified as positive if any acquired view yielded a positive prediction. To address the variability in performance across the multiple architectures evaluated, we reported the mean and standard deviation (Mean ± SD) of the performance metrics across all 5 folds. Furthermore, to assess whether the observed differences between the top-performing model and the other baseline models were statistically significant, we conducted a corrected resampled paired *t*-test (27) with Holm-Bonferroni correction to rigorously account for the overlapping training data inherent in cross-validation. A *p*-value of less than 0.05 was considered statistically significant.

The segmentation loss function combines the Dice loss and the multi-class cross-entropy loss, defined in Equations 4 and 5, with equal weights as shown in Equation 6. In this study, the Dice loss was calculated separately for each class (including the background), and their average was used as the final Dice loss. The classification loss function employed the binary cross-entropy loss as shown in Equation 7. The formulas for the loss functions are as follows:


$$\begin{array}{cc} L_{\text{Dice}} & =\frac{1}{C}\overset{C-1}{\underset{c=0}{\sum }}\left(1-\frac{2\underset{i}{\sum }p_{i}^{\left(c\right)}g_{i}^{\left(c\right)}+\varepsilon }{\underset{i}{\sum }p_{i}^{\left(c\right)}+\underset{i}{\sum }g_{i}^{\left(c\right)}+\varepsilon }\right), \end{array}$$ (4)

$$\begin{array}{cc} L_{\text{CE}} & =-\frac{1}{N}\overset{N}{\underset{i=1}{\sum }}\overset{C-1}{\underset{c=0}{\sum }}g_{i}^{\left(c\right)}\log p_{i}^{\left(c\right)}, \end{array}$$ (5)

$$\begin{array}{cc} L_{\text{seg}} & =0.5L_{\text{Dice}}+0.5L_{\text{CE}}, \end{array}$$ (6)

$$\begin{array}{cc} L_{\text{cls}} & =-\left[y\log\left(\hat{p}\right)+\left(1-y\right)\log\left(1-\hat{p}\right)\right]. \end{array}$$ (7)

Here, *C* denotes the total number of classes (*C* = 4 in this study), and *N* represents the total number of pixels. $p_{i}^{\left(c\right)}$ indicates the predicted probability for class *c* at pixel *i*, and $g_{i}^{\left(c\right)}$ is the corresponding ground-truth label in one-hot encoding. ε is a small positive constant introduced to prevent division by zero; in this study, we set ε = 10^−5^. For the classification task, *y* represents the true binary label, and $\hat{p}$ denotes the predicted probability from the model.

## 3 Results

### 3.1 Performance evaluation metrics

To comprehensively evaluate the performance of our proposed pipeline, we utilized standard classification metrics, including accuracy, precision, sensitivity (recall), specificity, F1-score, and the Area Under the Receiver Operating Characteristic curve (AUROC). These metrics provide a holistic view of the model's diagnostic capability in identifying craniosynostosis. For the segmentation task, the Dice Similarity Coefficient (DSC) was employed to quantify the spatial overlap between the predicted suture masks and the ground-truth annotations.

### 3.2 Segmentation results

Table 3 summarizes the segmentation performance of various models on the test set (*n* = 213). Due to the scarcity of samples for specific subtypes and the high granularity of the 16 diagnostic categories, we aggregated the evaluation into four primary diagnostic groups: Normal, Coronal, Sagittal, and Lambdoid. It is important to note that since a single patient may exhibit multiple suture fusions (e.g., combined coronal and sagittal synostosis), the sum of *n* for each suture category exceeds the total number of test images. Macro Dice represents the average Dice score calculated across the entire test set.

**Table 3:** Comparison of dice coefficients of segmentation models.

Model	Normal (*n* = 102)	Coronal (*n* = 59)	Sagittal (*n* = 58)	Lambdoid (*n* = 23)	Macro dice (*n* = 213)
U-Net	0.723	0.496	0.607	0.554	0.624
U-Net++	0.727	0.497	0.590	0.538	0.625
DeepLabV3	0.742	0.524	0.623	0.574	0.650
DeepLabV3+	0.734	0.526	0.608	**0.613**	0.641
nnU-Net	**0.767**	**0.528**	**0.624**	0.585	**0.665**
TransUNet	0.737	0.428	0.526	0.534	0.599

Bold values indicate the highest (best) segmentation performance in each column.

As shown in the results, nnU-Net achieved the highest Macro Dice score of 0.665 and demonstrated superior performance across most categories. To further validate this superiority, we analyzed the variability and statistical significance across the 5-fold cross-validation (see Supplementary Table 1). nnU-Net consistently outperformed all other baseline architectures with the highest average Macro Dice score (0.6768 ± 0.0247), and the corrected resampled paired t-test with Holm-Bonferroni correction confirmed that these performance differences were statistically significant (*p* < 0.01 for all comparisons). This can be attributed to nnU-Net's self-configuring framework, which automatically optimizes pre-processing, network topology, and training hyperparameters based on the dataset properties, thereby handling the variability of skull X-rays more effectively than fixed architectures. In contrast, TransUNet, a Transformer-based model, exhibited the lowest performance (0.599). Vision Transformers generally lack the inductive biases (e.g., translation invariance) inherent in CNNs and typically require large-scale datasets to learn effective spatial representations (21). Given the limited size of our medical dataset, the Transformer-based approach failed to generalize well-compared to CNN-based baselines.

Regarding the performance gap between diagnostic groups, the Normal cases consistently showed higher segmentation accuracy compared to the Craniosynostosis (CSO) groups. Specifically, Table 4 reveals a notably larger standard deviation for the CSO group (±0.241) compared to the Normal group (±0.122). This increased variance is largely attributable to the severe intra-class imbalance within the craniosynostosis subtypes. While major subtypes such as single-suture fusions are relatively well-represented, complex multi-suture synostoses are extremely rare in the dataset. Consequently, the model's segmentation capability fluctuates significantly depending on the rarity of the specific subtype, leading to a wider spread in performance metrics for the CSO group. Furthermore, while Normal skulls exhibit consistent anatomical structures, craniosynostosis cases present highly diverse and severe deformities, making the learning manifold significantly more complex.

**Table 4:** Comparison of dice coefficients for the nnU-Net model across different views and diagnostic groups.

Diagnosis	AP (*n* = 55)	Lateral (*n* = 129)	Towne's (*n* = 29)	Total (*n* = 213)
Normal (*n* = 102)	0.787 ± 0.097	0.771 ± 0.125	0.657 ± 0.108	0.767 ± 0.122
CSO (*n* = 111)	0.498 ± 0.226	0.658 ± 0.211	0.457 ± 0.251	0.571 ± 0.241
Average	0.613 ± 0.233	0.722 ± 0.177	0.505 ± 0.241	0.665 ± 0.217

Values are presented as Mean ± Standard Deviation.

**Figure 5:**
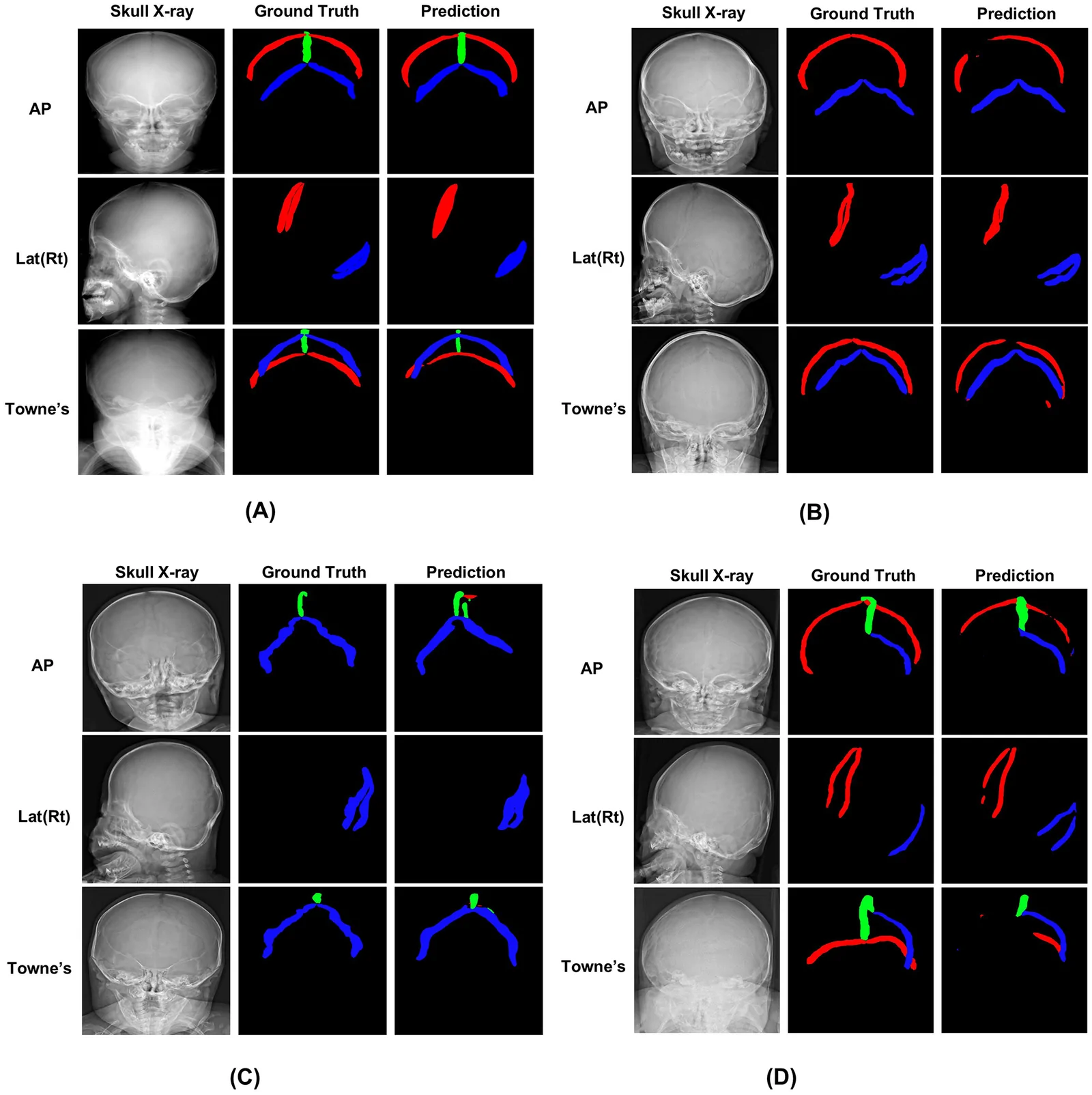
Qualitative segmentation results across different diagnostic types (Red: Coronal, Green: Sagittal, Blue: Lambdoid suture). **(A)** Normal. **(B)** Sagittal. **(C)** Coronal(Bi). **(D)** Lambdoid(Rt). Each panel displays three views of skull X-rays: AP, Lat(Rt), and Towne's from top to bottom. For each view, the input image, ground truth mask, and predicted mask are shown from left to right.

Figure 5 illustrates example segmentation results predicted by the model for (A) Normal, (B) Sagittal, (C) Coronal(Bi), and (D) Lambdoid(Rt) cases. Each panel displays three views of skull X-rays: AP, Lat(Rt), and Towne's from top to bottom. For each view, the input image, ground truth mask, and predicted mask are shown from left to right.

In Figure 5A, all three sutures are clearly visible in the AP view, and the predicted masks closely resemble the ground truth. In the AP views of the Sagittal craniosynostosis patient in Figure 5B and the Coronal(Bi) craniosynostosis patient in Figure 5C, the green sagittal suture and red coronal suture, respectively, are absent from both the ground truth and predicted masks. This can be interpreted as the model accurately reflecting the clinical characteristic that these sutures become unobservable due to craniosynostosis.

In the case of the Lambdoid(Rt) patient shown in Figure 5D, the model accurately predicted the unilateral visibility of the lambdoid suture in the AP view. However, in the Lat(Rt) view, the model erroneously predicted that both lambdoid sutures were visible. Furthermore, in the Towne's view, the segmentation performance was significantly degraded, often failing to accurately delineate the suture lines.

These instances highlight that segmentation results are susceptible to instability due to a combination of data scarcity and high anatomical complexity, where sutures are often entangled, indistinct, or exhibit low contrast against surrounding structures. This instability serves as the primary motivation for the introduction of the Mask-weighted 4-channel Input strategy.

### 3.3 Classification results

Table 5 presents the quantitative comparison of various classification models trained with the proposed method. Among the evaluated architectures, DenseNet-161 achieved the highest performance across key metrics, recording an Accuracy of 0.925 and an AUROC of 0.980. We also analyzed the fold-to-fold variability and statistical significance across the 5-fold cross-validation, as detailed in Supplementary Table 2. As shown, the observed differences between the top-performing DenseNet-161 and several other competitive models (e.g., ResNet, MobileNet, ViT, and Swin-T) were not statistically significant (n.s.). Crucially, the lack of significant variance across diverse backbones—ranging from lightweight CNNs to Transformer-based architectures—highlights the architectural robustness and stability of our proposed Mask-weighted 4-channel input framework. It implies that the consistent diagnostic performance stems primarily from our anatomically guided input representation itself, rather than relying on the capacity of a specific modern network. Based on these comprehensive results, DenseNet-161 was selected as the backbone network for subsequent ablation studies and performance evaluations.

**Table 5:** Quantitative performance comparison of various classification models.

Model	Accuracy	Precision	Sensitivity	Specificity	F1 score	AUROC
DenseNet-161	**0.925**	**0.915**	**0.915**	**0.933**	**0.915**	**0.980**
MobileNet	0.878	0.847	0.883	0.874	0.865	0.942
ResNet-18	0.911	0.903	0.894	0.924	0.898	0.966
ResNet-50	0.897	0.883	0.883	0.908	0.883	0.957
VGG-16	0.854	0.818	0.862	0.849	0.839	0.931
VGG-19	0.836	0.792	0.851	0.824	0.821	0.892
ViT	0.887	0.865	0.883	0.891	0.874	0.960
SwinT	0.826	0.771	0.862	0.798	0.814	0.912

Bold values indicate the highest classification performance in each column (achieved by DenseNet-161).

Table 6 compares the performance of different input strategies using the DenseNet-161 backbone. The “Base” method utilizes raw images without pre-processing, while the “Crop” method applies the text removal and skull ROI cropping pre-processing described in previous sections. “Proposed method” represents the Mask-weighted 4-channel input strategy. Although the “Base” model appears to show competitive performance, subsequent qualitative analysis (Grad-CAM) revealed that it primarily relies on non-anatomical artifacts, such as metadata text in the image corners, rather than the skull structure, rendering it clinically unreliable. The “Crop” method mitigates this by removing artifacts, but the proposed method achieved the significant highest performance (F1-score 0.915, AUROC 0.980), demonstrating that explicitly providing suture information leads to superior diagnostic accuracy.

**Table 6:** Comparison of performance between different input strategies using DenseNet-161.

Method	Accuracy	Precision	Sensitivity	Specificity	F1 score	AUROC
DenseNet-161 (Base)	0.901	0.869	0.915	0.891	0.891	0.978
DenseNet-161 (Crop)	0.901	0.884	0.894	0.908	0.889	0.953
**Proposed method**	**0.925**	**0.915**	**0.915**	**0.933**	**0.915**	**0.980**

Bold values indicate the highest classification performance in each column (achieved by the Proposed method).

Table 7 details the performance of the proposed model across different X-ray views. The model demonstrated strong separability in the AP and Towne's views, achieving an AUROC of 1.000. While the Specificity in the Towne's view was relatively low (0.429) due to a conservative decision threshold, an AUROC of 1.000 indicates that the model correctly ranks all positive cases higher than negative cases. The Lateral view also showed robust performance with an Accuracy of 0.915 and AUROC of 0.964.

**Table 7:** Performance evaluation of the DenseNet-161 model across different skull X-ray views.

View	Accuracy	Precision	Sensitivity	Specificity	F1 score	AUROC
AP (*n* = 55)	0.982	0.971	1.000	0.955	0.985	1.000
Towne's (*n* = 29)	0.862	0.846	1.000	0.429	0.917	1.000
Lateral (*n* = 129)	0.915	0.912	0.795	0.967	0.849	0.964

**Figure 6:**
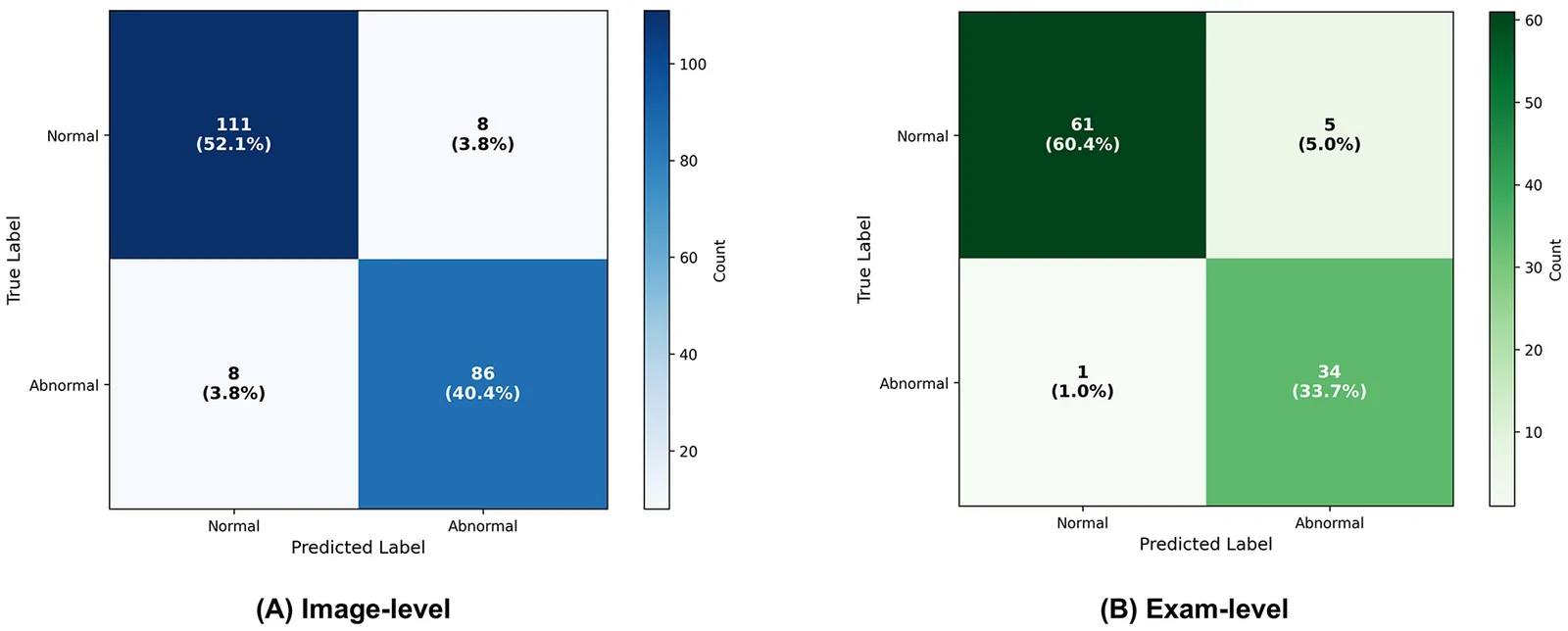
Confusion matrices for the diagnostic evaluations. **(A)** Image-level classification ensemble (*n* = 213). **(B)** Final exam-level diagnostic evaluation (*n* = 101 examinations from 74 patients).

Table 8 compares the diagnostic performance at the image level vs. the exam level. For exam-level diagnosis, we aggregated the predictions from all available views for each examination using a logical ‘OR' rule: an examination was diagnosed with craniosynostosis if at least one view was classified as positive. Conversely, an examination was considered normal only if all views were classified as negative. This comprehensive evaluation strategy significantly improved the diagnostic reliability, yielding an Exam-level Accuracy of 0.941 (95% CI: 0.89–0.98) and an AUROC of 0.994 (95% CI: 0.98–1.00), outperforming the single image-level analysis. To further validate these findings, Figure 6 visualizes the confusion matrices for (A) image-level and (B) exam-level evaluations. These matrices explicitly demonstrate the distribution of true positive and true negative predictions, visually confirming that the exam-level aggregation strategy effectively reduces misclassifications compared to the image-level approach.

**Table 8:** Comparison of classification performance: image-level vs. exam-level evaluation.

Evaluation level	Accuracy	Precision	Sensitivity	Specificity	F1 score	AUROC
Image-level Classification (*n* = 213)	0.925	**0.915**	0.915	**0.933**	0.915	0.980
**Exam-level evaluation** (*n* = 101)	**0.941**	0.872	**0.971**	0.924	**0.919**	**0.994**
(95% Confidence interval)	(0.89–0.98)	(0.75–0.97)	(0.91–1.00)	(0.85–0.98)	(0.84–0.98)	(0.98–1.00)

Bold values indicate the highest performance for each classification metric (column).

In actual clinical practice, evaluating multi-view skull radiographs is a standard approach for diagnosing craniosynostosis. According to established clinical guidelines, if there is any clinical suspicion or doubt regarding pathological fusion in the skull, the patient is promptly referred to a center of expertise or undergoes 3D-CT imaging to prevent missed diagnoses (3). To directly reflect this highly sensitive clinical workflow, we adopted the OR rule for exam-level aggregation. To empirically validate this clinical rationale, we evaluated various multi-view aggregation methods, as summarized in Table 9. While alternative aggregation methods, such as “Majority Voting” and the “AND” rule, achieved perfect Precision and Specificity, they suffered from a severe degradation in Sensitivity (0.743 and 0.600, respectively). For a computer-aided referral decision support tool, minimizing false negatives is paramount. The “OR” rule not only demonstrated the highest Sensitivity (0.971) but also achieved the highest overall Accuracy (0.941) and F1 Score (0.919) among the evaluated rules. Therefore, supported by both clinical guidelines and quantitative superiority, the OR rule proves to be the most suitable and most conservative aggregation strategy to assist clinical referral where missing a true positive case is highly detrimental.

**Table 9:** Comparison of exam-level multi-view aggregation strategies.

Aggregation method	Accuracy	Precision	Sensitivity	Specificity	F1 score	AUROC
**OR rule (any positive)**	**0.941**	0.872	**0.971**	0.924	**0.919**	0.994
Majority Voting (≥ 50% positive)	0.911	**1.000**	0.743	**1.000**	0.853	**0.996**
AND Rule (all positive)	0.861	**1.000**	0.600	**1.000**	0.750	**0.996**

Bold values indicate the highest performance for each metric (column) among the aggregation strategies.

In addition, we conducted an ablation study to evaluate the impact of different radiographic view combinations on the OR rule's performance, as detailed in Supplementary Table 3. The analysis revealed that the inclusion of the Towne's view marginally decreased specificity due to a severe data imbalance (predominantly CSO cases in Towne's projections). However, the model achieved excellent performance using only the AP and Lateral views (Sensitivity 0.971, Specificity 0.955), which are most commonly taken in general clinical practice.

Finally, to evaluate the model's diagnostic consistency across various developmental stages, we conducted an age-stratified performance analysis based on the exam-level OR rule. As summarized in Table 10, the proposed model maintained high diagnostic accuracy across all age groups. Notably, within the clinically critical early evaluation window of 0–12 months (*n* = 41), the model successfully identified all true craniosynostosis cases, yielding a Sensitivity of 1.000 and an AUROC of 1.000. Although the limited sample size in this specific infant demographic necessitates cautious interpretation, these preliminary results demonstrate robust diagnostic performance within our current dataset.

**Table 10:** Age-stratified performance analysis based on exam-level OR rule.

Age group	n	CSO	Normal	Accuracy	Sensitivity	Specificity	AUROC
0–6 months	24	19	5	0.9583	1.0000	0.8000	1.0000
7–12 months	17	11	6	0.9412	1.0000	0.8333	1.0000
**0–12 months (combined)**	**41**	**30**	**11**	**0.9512**	**1.0000**	**0.8182**	**1.0000**
13–24 months	10	2	8	1.0000	1.0000	1.0000	1.0000
25–60 months	34	3	31	0.9412	0.6667	0.9677	0.9785
≥ 61 months	16	0	16	N/A (single class)			

Bold values indicate the combined performance of the 0–12 months age group, highlighting the clinically critical early evaluation window.

### 3.4 Qualitative analysis via Grad-CAM

**Figure 7:**
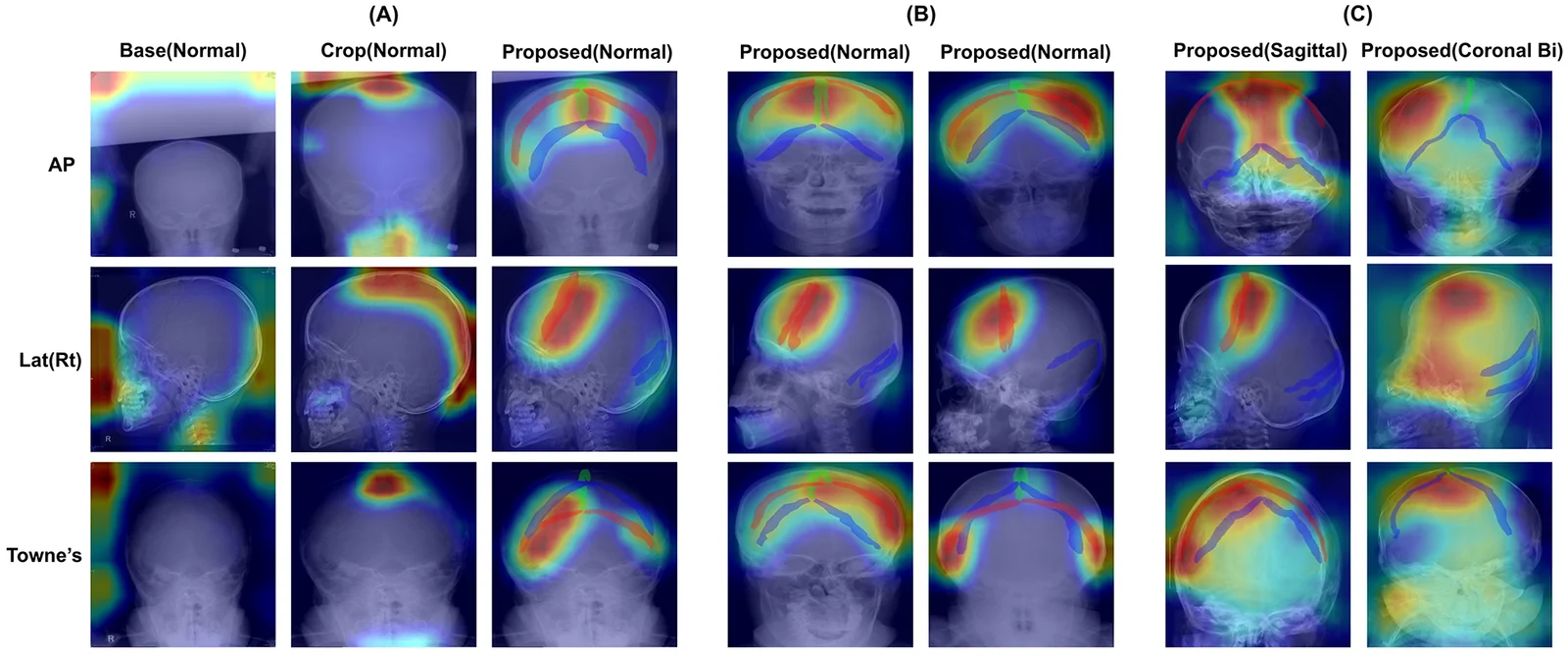
Grad-CAM visualization results comparing input strategies and diagnostic types. **(A)** Comparison of the “Base,” “Crop,” and “Proposed” strategies on a single Normal case. **(B)** Demonstration of the proposed method on additional Normal cases. **(C)** Patients with Sagittal and Coronal(Bi) craniosynostosis.

Figure 7 visualizes the Grad-CAM results. Figure 7A compares the three input strategies (Base, Crop, Proposed) on the same patient to illustrate the shift in model attention. To facilitate analysis, the ground-truth suture masks are overlaid semi-transparently on the visualization results. All presented cases correspond to instances where the classification model made correct predictions.

In the results for the “Base” model (DenseNet-161 without pre-processing), attention is erroneously concentrated on non-anatomical regions, specifically metadata text such as hospital names located in the image corners. This indicates that the model bases its predictions on irrelevant artifacts rather than anatomical features, rendering its decision-making process clinically unreliable.

The ‘Crop' model shows that pre-processing successfully shifts the attention to the skull region. However, the focus tends to fixate on the outer contours of the skull rather than the internal suture lines. This suggests that simple pre-processing alone is insufficient for the classification model to effectively recognize crucial suture features, limiting its diagnostic precision.

In contrast, the proposed method demonstrates that the model's attention is predominantly directed toward the suture regions during prediction. Notably, the attention is predominantly directed toward the coronal and sagittal sutures across all views. This behavior is interpreted as a result of the dataset distribution, where Sagittal and Coronal(Bi) craniosynostosis constitute the majority of positive cases, making these sutures critical decision boundaries for distinguishing between normal and pathological states.

A distinct pattern emerges when comparing Normal and CSO cases. In Normal cases, supported by high segmentation accuracy, the proposed model exhibits attention focused on the distinct suture lines. Conversely, for CSO cases, where suture segmentation performance is relatively lower (as shown in Table 4) due to fusion or faintness, the Grad-CAM activation maps tend to spread more broadly around the sutures or extend to adjacent cranial regions. This suggests that when suture features are obliterated or indistinct, the model does not rely solely on the imperfect mask information but compensates by utilizing secondary signs, such as morphological cranial deformations, from the original image channel. This validates the effectiveness of our 4-channel design strategy, which contributes to diagnostic robustness by providing the original radiographic information alongside the mask-weighted guidance.

As highlighted in the Introduction, differentiating craniosynostosis from non-pathological conditions such as positional plagiocephaly is a critical clinical challenge, as both conditions share similar morphological characteristics. To verify whether the proposed model relies on global shape deformation or correctly adheres to the primary pathology (suture fusion), we conducted an additional evaluation on five patients with positional plagiocephaly. Crucially, these images were collected from independent clinical cases that were completely excluded from the model development phase and were processed without any ground-truth labels.

**Figure 8:**
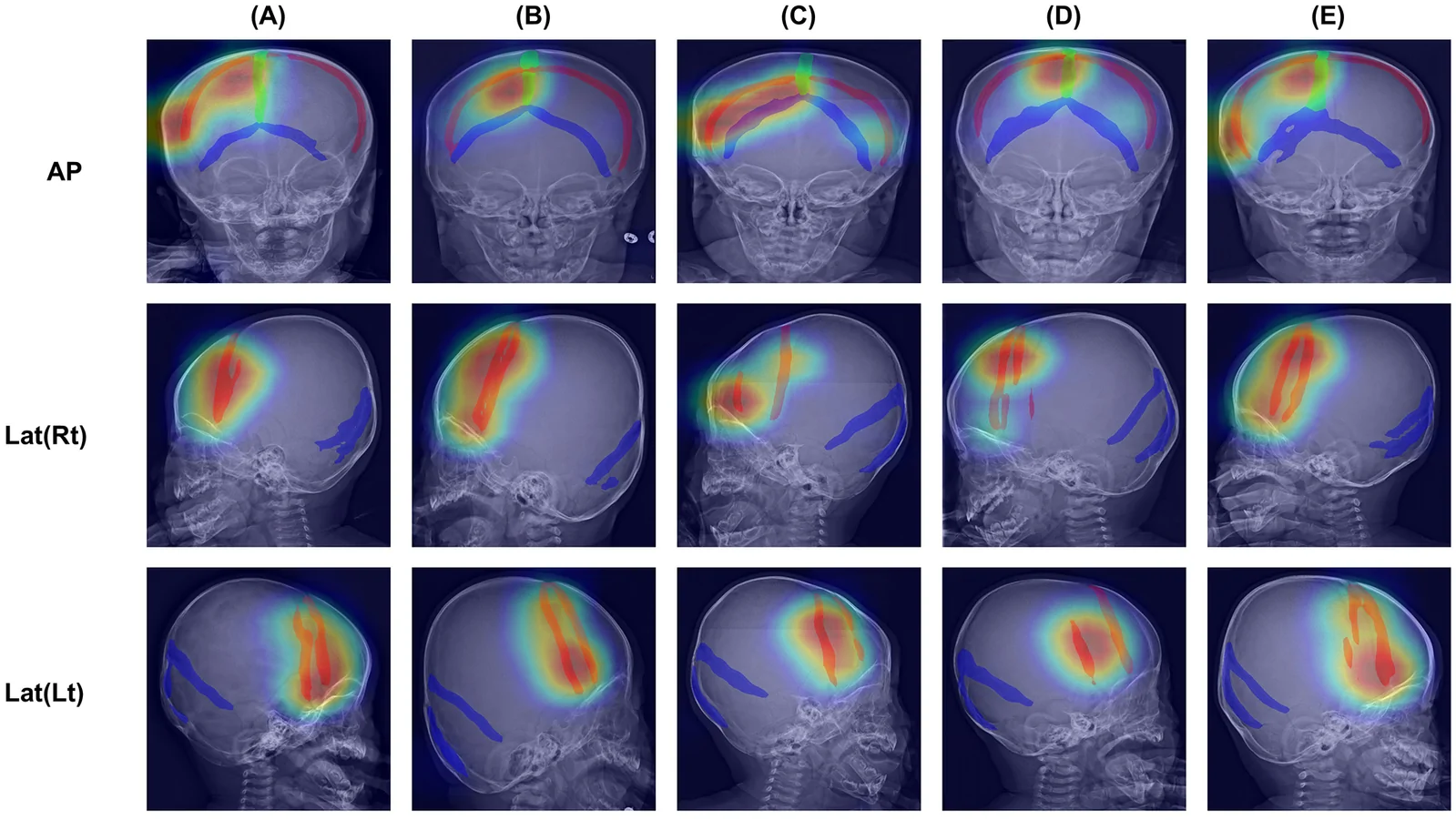
Visualization of segmentation and Grad-CAM results on five patients with Positional Plagiocephaly, whose images were completely excluded from model development. **(A–E)** Patient cases 1–5, respectively. The model maintains its focus on the sutures despite cranial deformation, confirming robustness against non-pathological anomalies.

Figure 8 presents the visualization results, where the predicted suture masks are overlaid with the Grad-CAM activation maps. Despite the significant cranial deformation observable in these cases, the proposed segmentation module successfully delineated the sutures. Furthermore, the Grad-CAM results show that the model's attention is focused on these suture lines, showing a pattern highly similar to that of the Normal cases in Figure 7. This indicates that the model is not misled by the secondary skeletal deformities caused by positional factors, but rather its attention correlates with the suture regions. Consequently, this result supports the anatomical validity of our method in differentiating true craniosynostosis from benign deformities, as it identifies the direct cause of the pathology rather than relying on ambiguous morphological shapes.

## 4 Discussion

In this study, we proposed a novel deep learning framework that integrates suture segmentation with a mask-weighted classification pipeline to enhance the diagnostic accuracy and anatomically valid evaluation of craniosynostosis using skull X-ray images. The most significant clinical implication of our findings lies in the potential to aid early detection in primary care settings. Craniosynostosis is a rare condition where the timing of treatment is critical (2); however, in many cases, patients are referred to tertiary hospitals only after the cranial deformation has significantly progressed. Therefore, timely clinical evaluation of suspected craniosynostosis at primary healthcare facilities using accessible modalities like skull radiography is essential (3). Yet, interpreting these X-rays is highly subjective and challenging for general practitioners. Our model addresses this gap by explicitly focusing on the primary pathology—suture fusion (11)—rather than ambiguous secondary deformations often targeted by previous deep learning models (4, 5). As supported by our Grad-CAM analysis, this capability suggests that the model could reliably assist in differentiating true craniosynostosis from morphologically similar non-pathological conditions like positional plagiocephaly (10).

While recent advancements have introduced various radiation-free diagnostic modalities, each presents specific clinical trade-offs that highlight the continued necessity of our approach. For instance, three-dimensional stereophotogrammetry (3DSPG) (7, 28) and 2D photograph-based deep learning models (8) offer non-invasive screening; however, they inherently rely on secondary external cranial deformations and cannot directly evaluate the internal status of the cranial sutures. Alternatively, advanced MRI sequences and ultrasonography provide comprehensive radiation-free visualization of the cranial anatomy (6). Yet, their widespread adoption as primary evaluation tools is often hindered by high costs, the need for specialized equipment, operator dependence, and, in the case of pediatric MRI, the frequent requirement for sedation. In this context, our proposed X-ray-based framework occupies a crucial clinical middle ground. It significantly minimizes radiation exposure compared to standard CT scans while directly extracting the primary pathological feature—suture fusion—thereby offering a highly accessible, cost-effective, and anatomically robust decision support tool to assist clinical referral in primary care settings.

Despite these promising clinical implications, this study possesses several limitations that must be explicitly addressed in future research. A notable limitation of this study is the age imbalance between the normal group (mean age: 59.8 ± 32.9 months) and the craniosynostosis group (mean age: 15.0 ± 18.5 months). This disparity reflects the inherent epidemiological and social characteristics encountered in real-world clinical practice. Specifically, parents often express significant anxiety and lack of knowledge regarding radiation exposure, leading to a strong reluctance to subject healthy infants to X-ray imaging (13, 29, 30). Consequently, normal pediatric skull X-rays are predominantly acquired only when children are older and more active, typically following incidents such as head trauma. Conversely, despite the ideal surgical window being 3–6 months (2), infants with craniosynostosis often present later because parents without medical expertise may initially attribute abnormal head shapes to simple positional molding or rely on unverified non-surgical interventions, such as helmet therapy, delaying visits to primary care and subsequent referral to tertiary hospitals. Because craniosynostosis has a low incidence of approximately 1 in 2,000 live births, collecting a large, age-matched normal infant cohort is inherently challenging. In the age-stratified analysis, the number of normal infants within the 0–6 month group was limited (*n* = 5), which constrains the statistical robustness of the specificity estimate for this subgroup. Future studies incorporating larger datasets of young normal infants are needed to comprehensively validate model performance within the early clinical evaluation window.

Furthermore, the current methodology relies on a two-stage framework where the segmentation and classification models are trained independently. This disjointed approach limits the full utilization of synergistic feature interactions; transitioning to an end-to-end joint training architecture could optimize these representations. Also, achieving pixel-perfect segmentation is inherently constrained by the nature of the imaging modality. Although ground-truth annotations were carefully performed by board-certified pediatric plastic surgeons cross-referencing CT scans, the fine and overlapping nature of cranial sutures on 2D X-rays introduces inevitable labeling uncertainty. Third, this study is primarily based on a retrospective, single-center dataset, which inherently limits the immediate generalizability of the findings. Although we provided preliminary evidence of clinical robustness by successfully evaluating the model on unseen hold-out cases of positional plagiocephaly (Figure 8) that were excluded from the training phase, comprehensive multi-center external validation is necessary prior to widespread clinical deployment. Finally, a class imbalance persists in rare subtypes and the Towne's view. Although the scarcity of normal Towne's projections caused a data-driven bias toward predicting pathology, the model demonstrates robust diagnostic support capabilities using standard AP and Lateral views.

To overcome these limitations, future research will focus on developing an end-to-end joint training architecture and acquiring multi-center datasets to rebalance minority subsets. With continued methodological refinements, we anticipate that this deep learning framework will evolve into a valuable clinical decision support system, ultimately contributing to safer, more accessible, and earlier referral of pediatric cranial deformities.

## Data Availability

The datasets presented in this article are not readily available because the datasets analyzed during the current study are not publicly available and cannot be shared due to the highly sensitive nature of the clinical data and strict patient privacy restrictions mandated by the Institutional Review Board (IRB). Requests to access the datasets should be directed to Hyeyeon Kwon, kwon14325@snu.ac.kr.
