# Supplementary material for: Deep learning-based craniosynostosis classification via suture segmentation and mask-weighted classification
Source: Front Med (Lausanne). 2026 Aug 14;13:1871961. doi: 10.3389/fmed.2026.1871961 (PMC13521908; doi:10.3389/fmed.2026.1871961)
Supplement: Supplementary file 1 [file Data_Sheet_1.pdf]

Supplementary Table 1: 5-Fold Cross-Validation Results for Suture Segmentation (Macro Dice). Statistical differences were assessed using the Corrected Resampled paired t-test with Holm-Bonferroni correction against the best-performing model (nnU-Net). ** $p < 0.01$.

Model	Fold 1	Fold 2	Fold 3	Fold 4	Fold 5	Mean ± SD	p-value (Holm)
**nnU-Net** ⋆	0.6347	0.6883	0.6764	0.6976	0.6872	**0.6768 ± 0.0247**	—
DeepLabV3	0.6280	0.6781	0.6711	0.6895	0.6813	0.6696 ± 0.0242	0.0053 **
DeepLabV3+	0.6252	0.6758	0.6683	0.6867	0.6783	0.6669 ± 0.0242	0.0034 **
U-Net	0.6139	0.6679	0.6577	0.6723	0.6703	0.6564 ± 0.0244	0.0031 **
U-Net++	0.6021	0.6627	0.6555	0.6692	0.6657	0.6510 ± 0.0278	0.0034 **
TransUNet	0.5847	0.6482	0.6244	0.6413	0.6490	0.6295 ± 0.0269	0.0034 **

Supplementary Table 2: 5-Fold Cross-Validation Results for Craniosynostosis Classification (Accuracy). Statistical differences were assessed using the Corrected Resampled paired t-test with Holm-Bonferroni correction against the best-performing model (DenseNet-161). n.s. indicates not significant.

Model	Fold 1	Fold 2	Fold 3	Fold 4	Fold 5	Mean ± SD	p-value (Holm)
**DenseNet-161** ⋆	0.8981	0.9276	0.9408	0.9107	0.9500	**0.9254 ± 0.0213**	—
ResNet-18	0.8889	0.9145	0.9211	0.9167	0.9250	0.9132 ± 0.0142	0.464 (n.s.)
ResNet-50	0.8611	0.9145	0.9276	0.9048	0.9250	0.9066 ± 0.0270	0.464 (n.s.)
MobileNet-V2	0.8565	0.8947	0.8947	0.8869	0.9375	0.8941 ± 0.0289	0.183 (n.s.)
ViT	0.8519	0.9408	0.8618	0.8333	0.8625	0.8701 ± 0.0413	0.464 (n.s.)
VGG-16	0.8148	0.9276	0.8092	0.8690	0.8313	0.8504 ± 0.0491	0.464 (n.s.)
Swin-T	0.8333	0.8947	0.8224	0.7500	0.9062	0.8413 ± 0.0629	0.464 (n.s.)
VGG-19	0.8056	0.5395	0.8355	0.7560	0.6312	0.7136 ± 0.1247	0.464 (n.s.)

Supplementary Table 3: Detailed analysis of exam-level performance across different radiographic view combinations and the impact of Towne's view class imbalance.

(A) Exam-level Performance by View Combinations using the OR Rule (Test Set, $n = 101$)

View Combination	n	Accuracy	Precision	Sensitivity	Specificity	F1 Score	AUROC	False Positives (FP)
AP only	55	0.9636	0.9429	1.0000	0.9091	0.9706	1.0000	2
Lateral only	97	0.9691	0.9677	0.9375	0.9846	0.9524	0.9990	1
AP + Lateral (Excluding Towne's)	101	0.9604	0.9189	0.9714	0.9545	0.9444	0.9983	3
AP + Lateral + Towne's (All views)	101	0.9406	0.8718	0.9714	0.9242	0.9189	0.9987	5

(B) Severe Class Imbalance of Towne's View Across the Dataset

Data Split	Normal Towne's Images	CSO Towne's Images	Proportion of Normal (%)
Total Dataset ($n = 1088$)	37	123	23.1%
Training/Validation Set ($n = 875$)	30	101	22.9%
Test Set ($n = 213$)	7	22	24.1%
